# Supplementary material for: Comparison of Performance between Single- and Multiparameter Luminescence Thermometry Methods Based on the Mn5+ Near-Infrared Emission
Source: Sensors (Basel). 2023 Apr 9;23(8):3839. doi: 10.3390/s23083839 (PMC10143882; doi:10.3390/s23083839)
Supplement: Supplementary file 1 [file sensors-23-03839-s001.zip › sensors-2279352-supplementary.pdf]

SUPPORTING INFORMATION

Comparison of Performance between Single- and Multiparameter Luminescence Thermometry Methods Based on the Mn^{5+} Near-Infrared Emission

Tahani A. Alrebdi [1], Abdullah N. Alodhayb [2], Zoran Ristić [3] and Miroslav D. Dramićanin [3,*]

[1] Department of Physics, College of Science, Princess Nourah Bint Abdulrahman University, P.O. Box 84428, Riyadh 11671, Saudi Arabia
[2] Department of Physics and Astronomy, College of Science, King Saud University, P.O. Box 2455, Riyadh 11451, Saudi Arabia
[3] Centre of Excellence for Photoconversion, Vinča Institute of Nuclear Sciences—National Institute of the Republic of Serbia, University of Belgrade, P.O. Box 522, 11001 Belgrade, Serbia
* Correspondence: dramican@vinca.rs

1. Scanning Electron Microscopy

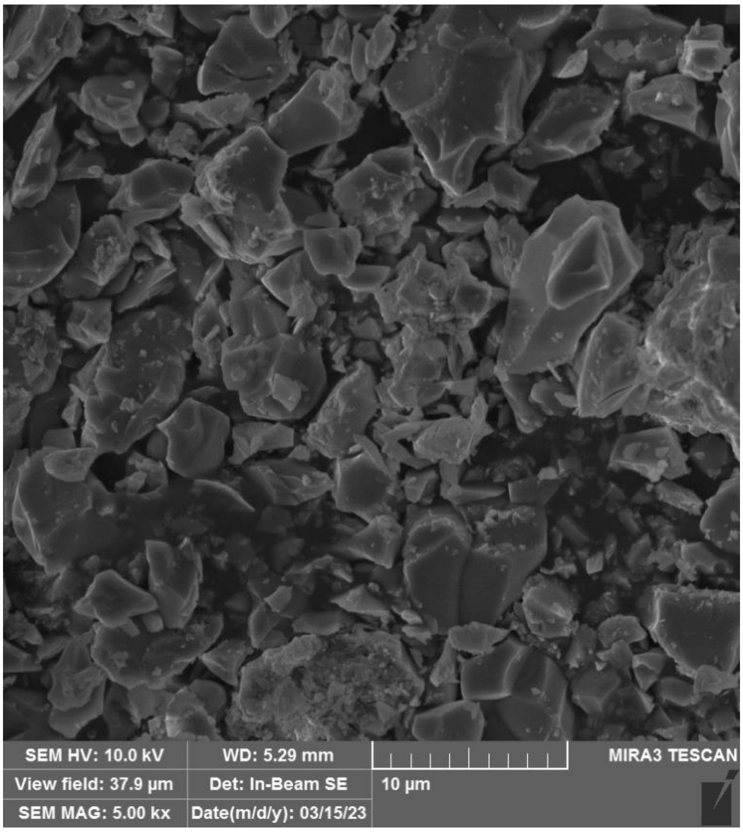

Figure S1. Scanning electronic microscopy image of $Ca_6BaP_4O_{17}$:(0.75%)Mn^{5+} near-infrared phosphor powder. Phosphor is composed of micron-size particles of irregular shape.

2. Powder X-ray Diffraction

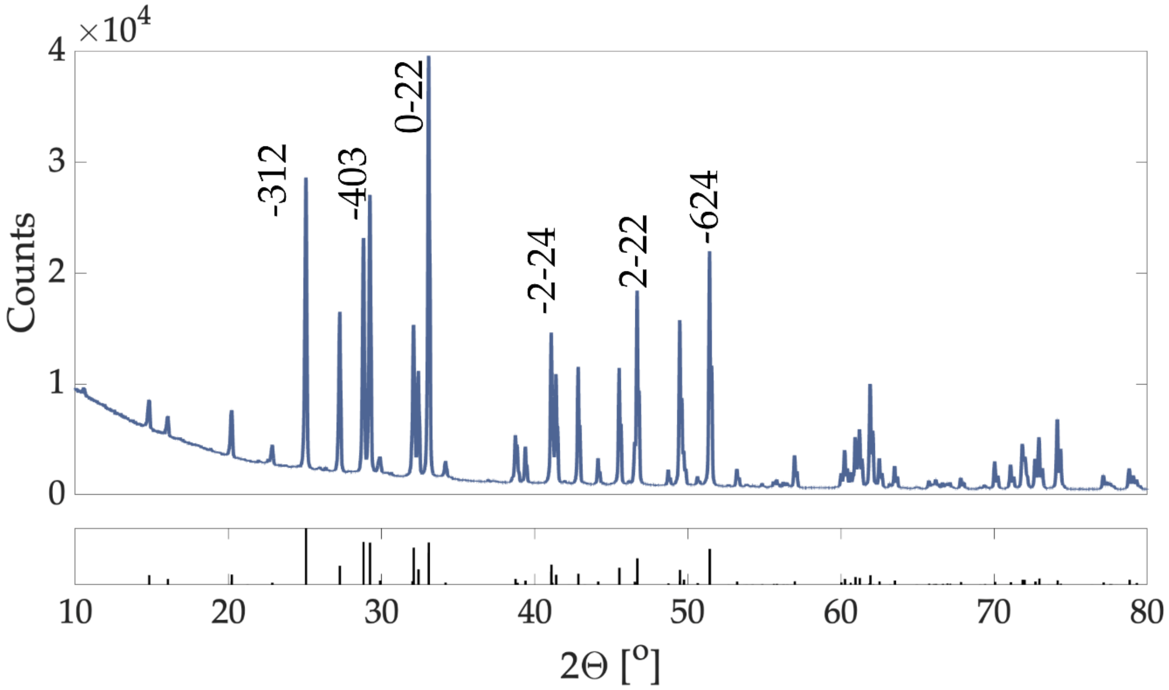

Figure S2. Powder X-ray diffractogram of $Ca_6BaP_4O_{17}$:(0.75%)Mn^{5+} near-infrared phosphor powder. Reflection peaks are indexed in accordance with data presented in the reference [Dramićanin, M.D.; Marciniak, Ł.; Kuzman, S.; Piotrowski, W.; Ristić, Z.; Periša, J.; Evans, I.; Mitrić, J.; Đorđević, V.; Romčević, N.; Brik, M.G.; Ma, C.-G. Mn^{5+}-activated $Ca_6Ba(PO_4)_4O$ near-infrared phosphor and its application in luminescence thermometry. *Light Sci. Appl.* **2022**, *11*, 279-292.].

3. Background Subtraction for the Evaluation of Anti-Stokes and Stokes Emission Intensities

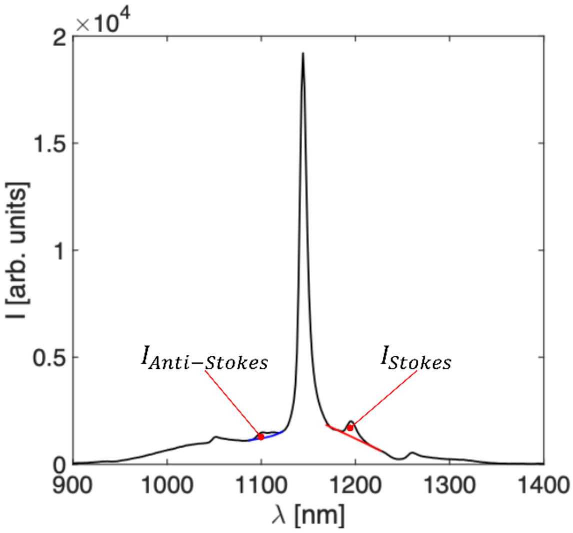

Figure S3. The removal of background emission from Stokes and anti-Stokes emission sidebands. To analyze the temperature behavior of Stokes and Anti-Stokes peaks the background is removed. This was done by fitting the third order polynomials on the emission data in the vicinity of the corresponding peaks. The values of such obtained polynomials inside the corresponding peaks were than used as a background.
